# Evolution of developmental roles of *Pax2/5/8 *paralogs after independent duplication in urochordate and vertebrate lineages

**DOI:** 10.1186/1741-7007-6-35

**Published:** 2008-08-22

**Authors:** Susan Bassham, Cristian Cañestro, John H Postlethwait

**Affiliations:** 1 Center for Ecology and Evolutionary Biology, University of Oregon, Eugene, OR, 97403, USA; 2 Institute of Neuroscience, University of Oregon, Eugene, OR, 97403, USA

## Abstract

**Background:**

Gene duplication provides opportunities for lineage diversification and evolution of developmental novelties. Duplicated genes generally either disappear by accumulation of mutations (nonfunctionalization), or are preserved either by the origin of positively selected functions in one or both duplicates (neofunctionalization), or by the partitioning of original gene subfunctions between the duplicates (subfunctionalization). The Pax2/5/8 family of important developmental regulators has undergone parallel expansion among chordate groups. After the divergence of urochordate and vertebrate lineages, two rounds of independent gene duplications resulted in the *Pax2, Pax5*, and *Pax8 *genes of most vertebrates (the sister group of the urochordates), and an additional duplication provided the *pax2a *and *pax2b *duplicates in teleost fish. Separate from the vertebrate genome expansions, a duplication also created two *Pax2/5/8 *genes in the common ancestor of ascidian and larvacean urochordates.

**Results:**

To better understand mechanisms underlying the evolution of duplicated genes, we investigated, in the larvacean urochordate *Oikopleura dioica*, the embryonic gene expression patterns of *Pax2/5/8 *paralogs. We compared the larvacean and ascidian expression patterns to infer modular subfunctions present in the single pre-duplication *Pax2/5/8 *gene of stem urochordates, and we compared vertebrate and urochordate expression to infer the suite of *Pax2/5/8 *gene subfunctions in the common ancestor of olfactores (vertebrates + urochordates). Expression pattern differences of larvacean and ascidian Pax2/5/8 orthologs in the endostyle, pharynx and hindgut suggest that some ancestral gene functions have been partitioned differently to the duplicates in the two urochordate lineages. Novel expression in the larvacean heart may have resulted from the neofunctionalization of a *Pax2/5/8 *gene in the urochordates. Expression of larvacean *Pax2/5/8 *in the endostyle, in sites of epithelial remodeling, and in sensory tissues evokes like functions of *Pax2*, *Pax5 *and *Pax8 *in vertebrate embryos, and may indicate ancient origins for these functions in the chordate common ancestor.

**Conclusion:**

Comparative analysis of expression patterns of chordate Pax2/5/8 duplicates, rooted on the single-copy *Pax2/5/8 *gene of amphioxus, whose lineage diverged basally among chordates, provides new insights into the evolution and development of the heart, thyroid, pharynx, stomodeum and placodes in chordates; supports the controversial conclusion that the atrial siphon of ascidians and the otic placode in vertebrates are homologous; and backs the notion that *Pax2/5/8 *functioned in ancestral chordates to engineer epithelial fusions and perforations, including gill slit openings.

## Background

Ohno's classical model to explain the fate of genes after gene duplication [1] predicts that one gene duplicate preserves the original gene function while its paralog either disappears by accumulation of detrimental mutations (called nonfunctionalization [2]) or occasionally acquires beneficial mutations that confer novel, positively selected functions (called neofunctionalization [2]). The duplication-degeneration-complementation (DDC) model predicts a third alternative, in which the two duplicate genes become permanently preserved as a consequence of complementary, degenerative mutations that result in partitioning of ancestral subfunctions, so that the sum of the functions of two paralogs equals the functions of the original gene prior to the duplication [2,3]. The DDC model also predicts that after the initial preservation of the two duplicates, whether by subfunctionalization or from neofunctionalization, further partitioning of redundant subfunctions may occur. Studies show that functional constraints on genes duplicated in whole-genome duplications are relaxed, compared with singletons, for tens of millions of years [4]. In addition, novel functions may originate over time, their evolution facilitated by the relaxation of pleiotropy occasioned by fewer tasks in each descendent gene duplicate compared with its single copy gene ancestor [2,5-9].

Understanding the evolution of duplicated genes is important because of the hypothesis that gene duplicates provide opportunities for the evolution of reproductive barriers that lead to lineage divergence [10], and for the origin of evolutionary novelties [1,11,12]. In vertebrates, many sets of paralogous genes arose in two rounds of genome duplication (R1 and R2) that took place in early vertebrate evolution [12-18]. An additional round of genome duplication (R3) occurred in the teleost lineage after ray-fin fish diverged from lobe-fin fish, and provided additional gene family members observed in many fish models [19-23]. It has been suggested that the R1 and R2 genome amplifications facilitated the origin of vertebrate innovations [24], and the R3 event may have facilitated the teleost species radiation [9,25].

Non-vertebrate chordates often have single copies of vertebrate gene families because their lineages diverged from the vertebrate lineage before the R1 and R2 genome duplication events. Recent phylogenomic analyses converge on the conclusion that the chordate subphylum Urochordata, which includes the classes Larvacea and Ascidiacea, are the closest living relatives of the vertebrates, constituting the group Olfactores (vertebrates + urochordates), while the subphylum Cephalochordata, including the amphioxus, diverged basally among chordates ([7,26-30], reviewed in [9]). As gene duplication is pervasive [31,32], non-vertebrate chordates sometimes have genes that duplicated independently in the cephalochordate [33-35] or urochordate lineages [35-41].

We propose that the comparative analysis of gene expression patterns in a gene family that experienced lineage-specific, independent duplication events, interpreted in a phylogenetic context and with respect to subfunction partitioning, can help in the inference of ancient gene functions and in the identification of gene functions that arose by neofunctionalization, and thus may be important for lineage divergence and the origin of developmental novelties. To test this proposition, we examined the *Pax2/5/8 *gene family. *Pax2/5/8 *genes encode transcription factors with conserved motifs, including a paired domain, homeodomain, and octapeptide, and are associated with mechanosensory development in mammals [42], frogs [43], fish [44,45], flies [46,47], ascidians [48] and in mollusks [49], while in a cnidarians, the apparent *Pax2/5/8 *homolog is associated with nerve and sensory cell differentiation [50,51].

*Pax2/5/8 *genes duplicated independently in different chordate lineages. Within the vertebrates, tetrapods have three members of the *Pax2/5/8 *gene family (*Pax2*, *Pax5 *and *Pax8*), and teleosts additionally have duplicate *pax2 *genes ([45] and references therein). In contrast to vertebrates, the basally diverging cephalochordate amphioxus possesses a single *Pax2/5/8 *gene that is equally related to *Pax2*, *Pax5 *and *Pax8 *[52], and urochordates have two *Pax2/5/8 *genes (*Pax2/5/8a *and *Pax2/5/8b*) [38,53,54] that originated in a duplication event prior to the divergence of larvacean and ascidian lineages [38]. The chordate *Pax2/5/8 *family embodies a full spectrum of gene evolutionary events: non-duplication (in amphioxus); independent duplications (within urochordates and vertebrates); gene loss (for example, tetrapods have just three of the four paralogs expected from two rounds of genome duplication); neofunctionalization (for example, vertebrate *Pax5 *in lymphocyte development [55]); and ancestral subfunctions appear to have been partitioned between paralogs within a lineage and, further, differently partitioned between lineages (for example, *Pax2 *and *Pax8 *in fish and mammal thyroids [56]).

The independent duplication of the stem olfactores' *Pax2/5/8 *gene in vertebrate and urochordate lineages provides replicate evolutionary experiments to explore the principles of subfunction partitioning and the origin of novel functions. To exploit this opportunity, we provide here a detailed description of expression patterns for *Pax2/5/8 *paralogs during development of the larvacean urochordate *Oikopleura dioica*. We then compare our results with expression patterns of *Pax2/5/8 *orthologs in ascidians, with independently duplicated *Pax2/5/8 *paralogs in vertebrates, and with the non-duplicated *Pax2/5/8 *gene in amphioxus as an outgroup. We discuss the expression of the *Pax2/5/8 *gene family during development of the heart, endostyle, pharynx, and sensory organs, and provide new insights that reconcile previous conflicting hypotheses about the homology of the ascidian atrial primordia and the vertebrate otic placode. This work thus illustrates the power of comparative analyses of independently duplicated genes to infer ancestral gene subfunctions, modules that can segregate independently from each other to evolving gene duplicates.

## Results

### Functional motif variation and gene structure of chordate *Pax2/5/8 *paralogs

**Figure 1:**
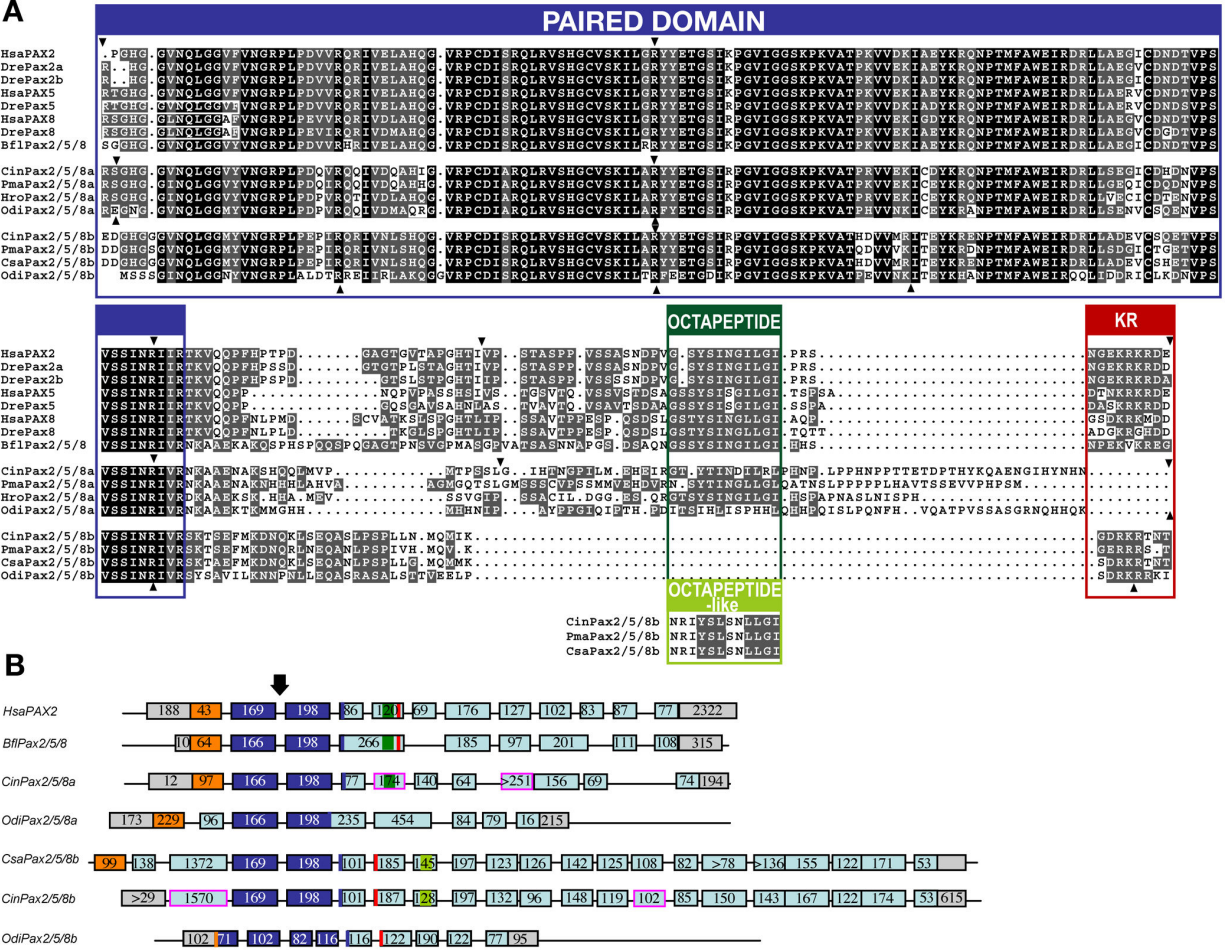
**Comparison of chordate *Pax2/5/8 *proteins and gene structures**. (A) Alignment of the chordate Pax2/5/8 proteins showing the conserved DNA-binding paired-domain (dark blue), the octapeptide motif (dark green), the octapeptide-like motif (light green), and the lysine-arginine (KR) rich region (red). Arrowheads indicate the positions of introns. (B) Exon-intron organization deduced from the comparison of ESTs and genomic regions available in public databases (Ghost: , JGI: , and NCBI: ). Numbers indicate the length of the exons (boxes) in base pairs. The position of the conserved domains shown in (A) is indicated with the same code of colors. Exons containing the putative beginning of the coding sequence are labeled in orange. Exons with low degree of sequence conservation and which are hardly alignable among different organisms are labeled in light blue. Exon regions containing 5' and 3'UTR are labeled in grey. Analysis of EST sequences suggested the presence of multiple splice variants, revealing exons (in pink) that were absent from other EST sequences for the same gene; these alternates include the exon harboring the poorly conserved octapeptide motif from *Ciona intestinalis*. The arrow indicates the totally conserved position of the intron within the paired domain. For a phylogenetic analysis of the chordate Pax2/5/8 proteins, see Additional file 1. Bfl: *Branchiostoma floridae*; Cin: *Ciona intestinalis*; Csa: *Ciona savignyi*; Dre: *Danio rerio*; Hro: *Halocynthia roretzi*; Hsa: *Homo sapiens*; Odi: *Oikopleura dioica*; Pma: *Phallusia mammillata*.

Pax2/5/8 proteins are transcription factors, whose sequence is poorly conserved across Chordata outside of two functional motifs: the paired domain, which interacts with the DNA of target genes (dark blue in Figure 1), and an octapeptide motif (dark green in Figure 1), which is conserved with other Pax proteins [57], functions in repression of Pax transactivation [58], and interacts with other transcriptional cofactors [59]. Sequence alignment of various chordate Pax2/5/8 proteins (Figure 1) reveals that while the sequence of the DNA-interacting paired domain is highly conserved across all chordate Pax2/5/8 proteins analyzed, the octapeptide motif is less conserved. In urochordates, for instance, the octapeptide is present in the ascidian *Halocynthia roretzi *[48], and, contrary to a previous report [60], our alignment reveals it is also present in *Ciona intestinalis *Pax2/5/8a (Figure 1A). The octapeptide sequence of the *Oikopleura Pax2/5/8a *gene is poorly conserved, and the octapeptide motif is absent from the expected position in all urochordate Pax2/5/8b proteins (Figure 1A). Interestingly, we have identified a new motif (light green in Figure 1B) that is conserved among all ascidian *Pax2/5/8b *paralogs, located further toward the carboxyl end. As this newly recognized sequence shows some similarities with the octapeptide motif, we have called it the 'octapeptide-like' motif (bottom Figure 1A). We could not identify an octapeptide-like motif in the *Oikopleura *Pax2/5/8b. Our alignment also reveals a lysine-arginine-rich domain (in red in Figure 1) that is conserved in vertebrate Pax2 and amphioxus Pax2/5/8, is variable among vertebrate Pax5 and Pax8 paralogs, and is present in urochordate Pax2/5/8b but not Pax2/5/8a. This protein motif marks the beginning of an amino acid range identified as important for greatly increasing vertebrate Pax2 protein's transactivation activity [58].

Comparison of gene structures revealed that, while most chordate *Pax2/5/8 *genes have 8 to 11 exons, ascidian *Pax2/5/8b *paralogs have 20 exons, which code for a protein of about 1300 amino acids, three times longer than the approximately 400 amino acid-long Pax2/5/8 proteins from other chordates. This difference in size is due mainly to an exon more than 1200 nucleotides long located upstream of the paired domain. Our analysis of publicly available EST sequences confirmed the presence of this large exon in gene models predicted for *Pax2/5/8b *in *Ciona intestinalis *(EMSBL ID ENSCINT00000012344) and in *Ciona savignyi *(ENSCSAVG00000001640) (data not shown). This large exon is absent from *O. dioica Pax2/5/8 *paralogs and may have evolved in the ascidian lineage after the divergence of urochordates.

In conclusion, various *Pax2/5/8 *paralogs appear to have lost ancestral features and evolved new structural motifs or new exons, as would be predicted by the DDC model applied to *Pax2/5/8 *paralogs evolving after independent gene duplication events. The identification of these structural features focuses attention on regions to test for function to learn the roles each motif may have played in the origin of lineage-specific morphologies.

### Developmental expression of the Oikopleura paralogs *Pax2/5/8a *and *Pax2/5/8b*

#### Tailbud stages

**Figure 2:**
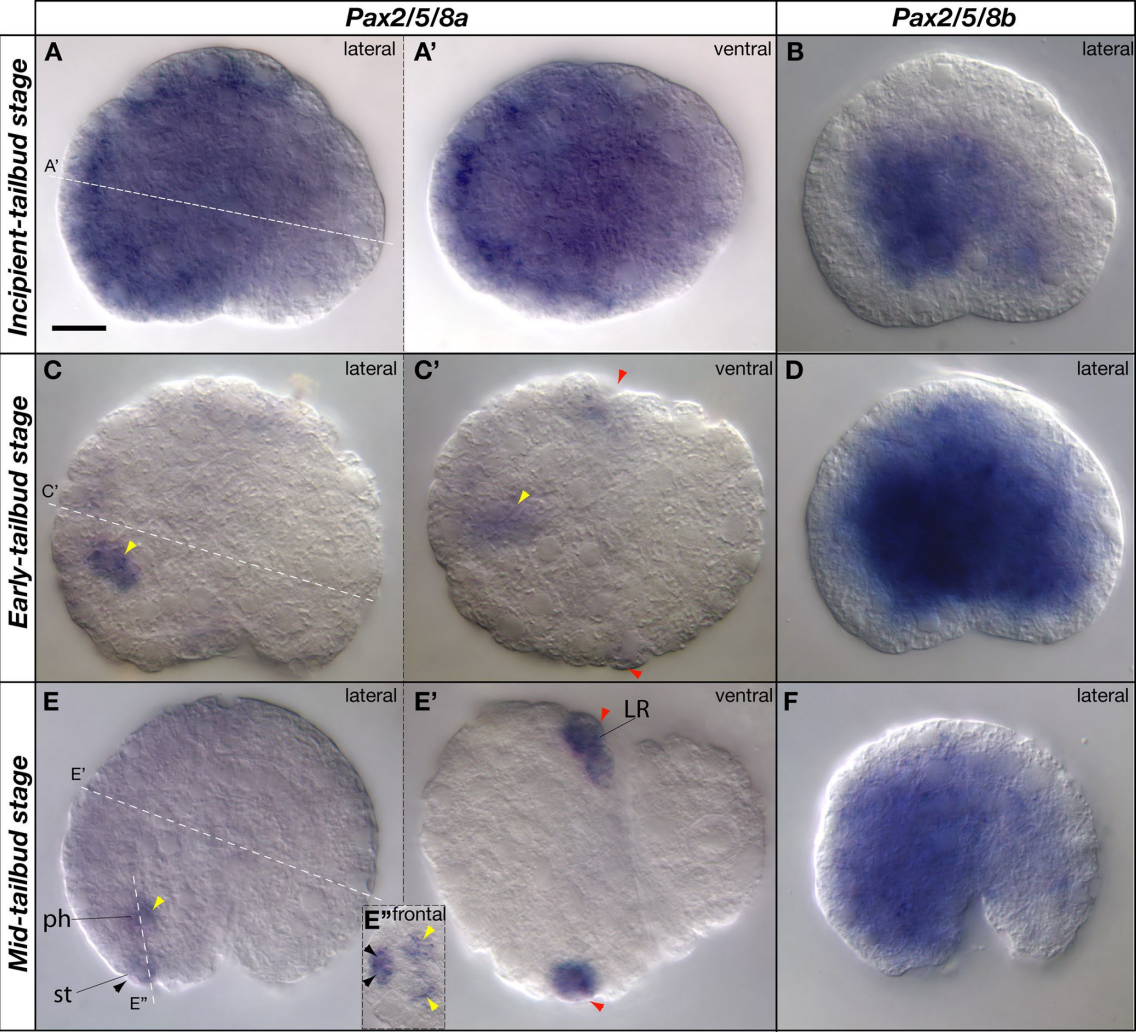
**Expression of *Pax2/5/8 *paralogs at tailbud stages in *Oikopleura dioica***. Whole mount in situ hybridization of *Pax2/5/8a *(A, C, E) and *Pax2/5/8b *(B, D, F) at incipient-tailbud stage (A, B), early-tailbud stage (C, D), and mid-tailbud stage (E, F) in lateral views (anterior to the left and dorsal to the top). Specific aspects of the expression domains are shown in ventral (A', C' and E') and frontal views (E") in optical sections in the plane of the dashed white lines. Colored arrowheads label expression domains (black, stomodeum, st; yellow, anterior pharynx, ph; red, placodal precursor of the Langerhans receptors, LR). Scale bar = 10 μm.

**Figure 3:**
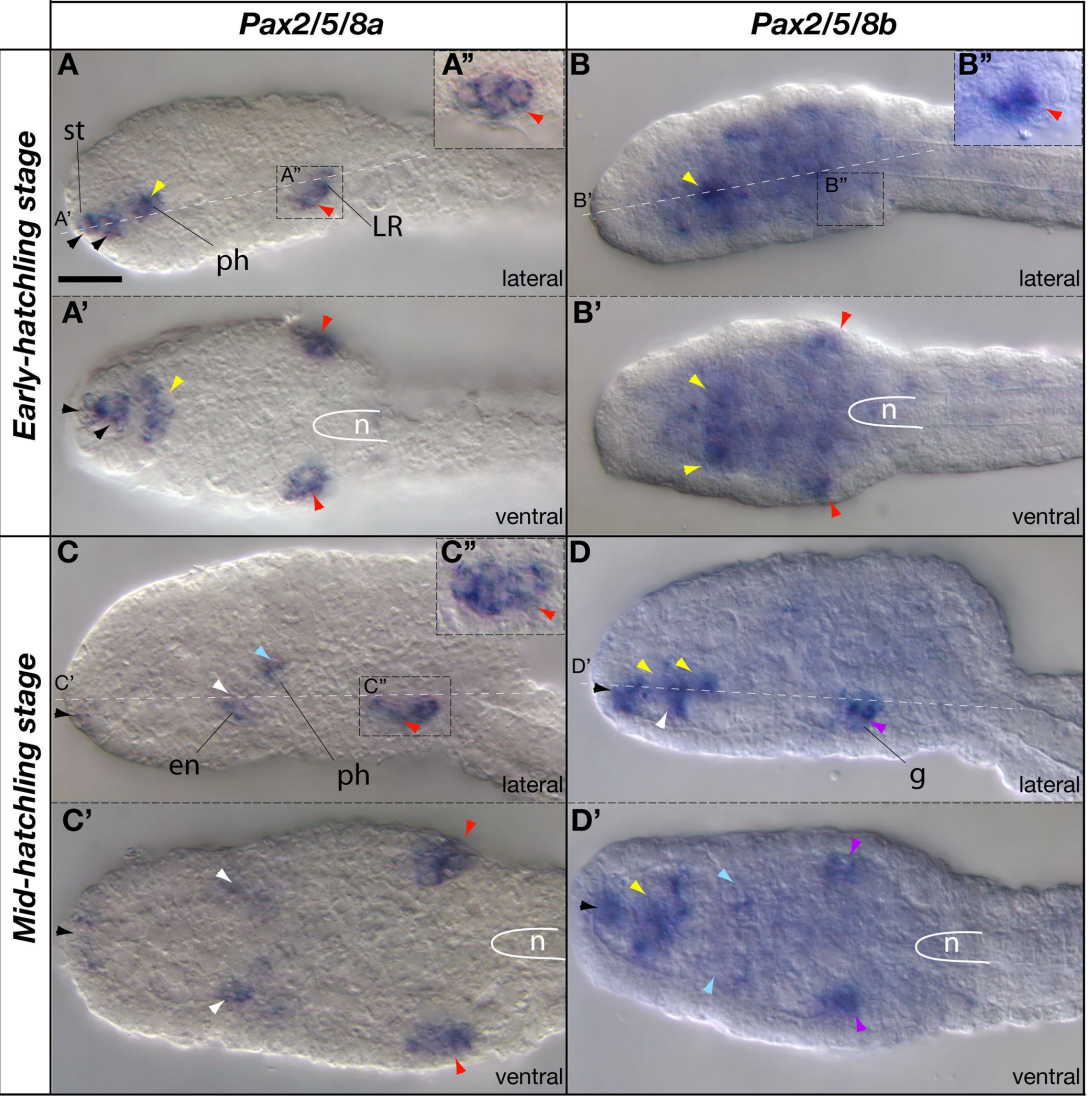
**Expression of *Pax2/5/8 *paralogs in *Oikopleura dioica *hatchlings**. Whole mount in situ hybridization of *Pax2/5/8a *(A, C) and *Pax2/5/8b *(B, D) in early-hatchling stage (A, B) and mid-hatchling stage (C, D). Lateral (A, B, C, D) and ventral (A', B', C' and D') views for each stage. Anterior is to the left and dorsal to the top. Insets (A", B", and C") show details of the regions on the surface of the embryo where the Langerhans receptors eventually form. Colored arrowheads label expression domains (black, stomodeum, st; yellow, anterior pharynx, ph; blue, posterior pharynx, ph; white, endostyle, en; purple, presumptive cell precursors of the gills, g; red, placodal precursor of the Langerhans receptors). The position of the anterior tip of notochord (n) is demarcated (white line). Scale bar = 10 μm.

At the end of gastrulation, *Pax2/5/8a *and *Pax2/5/8b *were both broadly expressed in the *Oikopleura *embryo. *Pax2/5/8a *was expressed mainly in the ectoderm of the trunk (Figure 2A), while *Pax2/5/8b *was expressed primarily in the interior of the trunk (Figure 2B). In early tailbud stages, *Pax2/5/8b *expression continued in its broad internal expression domain (Figure 2D), but *Pax2/5/8a *expression became restricted to two domains: a few medial cells at the boundary of the anterior brain and the pharynx (Figure 2C,C' yellow arrowheads), and a bilateral pair of ectodermal cells in the posterior trunk (Figure 2C,C' red arrowheads). By mid- and late-tailbud stage, the signal of the *Pax2/5/8a *expression domains strengthened (Figure 2E,E' yellow and red arrowheads), and a new domain appeared in the rostral-most ectoderm of the trunk (Figure 2E, E" black arrowhead), probably labeling the first two cells fated to invaginate into the stomodeum and form the oral epithelium (see below; Figure 3A).

#### Early hatchling and mid-hatchling stages

At the early hatchling stage, *Pax2/5/8a *expression maintained its late-tailbud stage pattern. The rostral-most expression domain in the stomodeal region expanded internally from the surface to form a group of contiguous internal cells (Figure 3A, black arrowheads), which were separated from the internal expression domain in presumptive pharynx/endostyle precursor cells (Figure 3A, yellow arrowhead). The bilateral *Pax2/5/8a *expression domain in the posterior part of the trunk included at least four ectodermal cells in the area where the Langerhans mechanoreceptors eventually develop (Figure 3A, red arrowheads), at a level slightly anterior to the tip of the notochord and adjacent to the homolog of the vertebrate hindbrain [38]. During early hatchling stages, the internal broad expression domain of *Pax2/5/8b *that was present at tailbud stages began to fade at the same time that two separate expression domains became distinct, one in the ectoderm at about the same bilateral position as the *Pax2/5/8a *ectodermal domain (Figure 3A', B', red arrowheads) and the other within the pharynx (Figure 3B, yellow arrowhead). Although *Pax2/5/8a *and *Pax2/5/8b *were both expressed in the ectoderm at the eventual position of the Langerhans receptors, close inspection revealed differences in the number and morphology of the *Pax2/5/8a *and *Pax2/5/8b *positive cells (Figure 3A", B"). By mid-hatchling stages, the bilateral *Pax2/5/8a *expression domain expanded to at least six ectodermal cells (Figure 3C"), while the brief flash of *Pax2/5/8b *expression in the ectoderm became undetectable (Figure 3C', D', purple arrowheads). Simultaneously, the distal portion of each gill pouch began to express *Pax2/5/8b*. It is not known if ectodermal cells contribute to the formation of the gill pouches at these early stages when the ectoderm is still forming its epithelial character.

**Figure 4:**
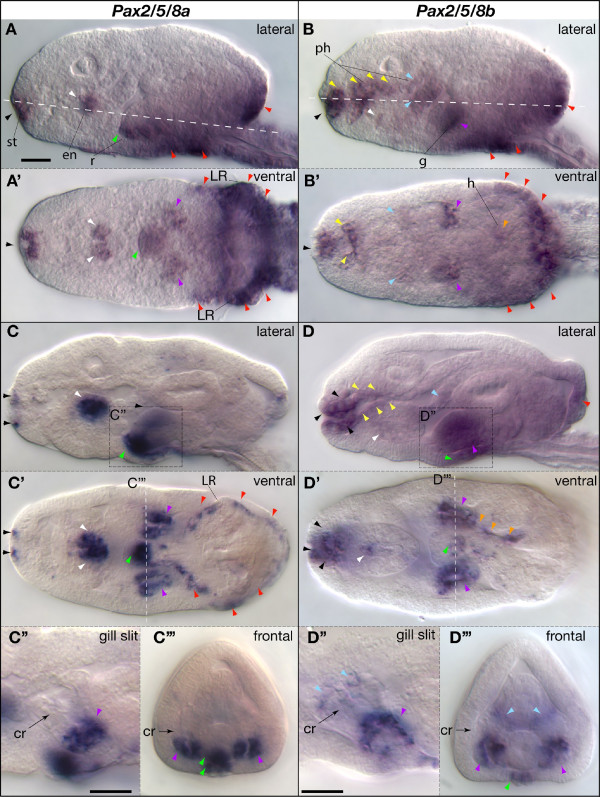
**Expression of *Oikopleura dioica Pax2/5/8 *paralogs during organogenesis**. Whole mount in situ hybridization of *Pax2/5/8a *(A, C) and *Pax2/5/8b *(B, D) in late hatchling stages during the initial (A, B) and advanced state (C, D) of the expansion of internal organ cavities during organogenesis. Lateral aspect (A, B, C and D) and integrated ventral view (A', B', C' and D') for each stage are shown. Anterior is to the left and dorsal to the top. Details of the *Pax2/5/8 *expression domains in the gills (dashed squares and lines in C and D) are shown in lateral (C" and D") and frontal (C"', D"') views. The position of the ciliary rings (cr) is labeled with an arrow. Colored arrowheads label some of the domains (black, stomodeum, st; yellow, anterior pharynx, ph; blue, posterior pharynx; white, endostyle, en; purple, gills, g; orange, medial wall of the heart, h; green, anus and rectum, r; red, base of the Langerhans receptors, LR, and posterior part of the trunk). Scale bar = 10 μm.

Expression of the two *Oikopleura Pax2/5/8 *paralogs during mid-hatchling stages appeared to be complementary in the pharynx in time and space: while *Pax2/5/8a *expression was diminishing in the stomodeum and the rostral pharynx (Figure 3C, C'), new *Pax2/5/8b *expression domains appeared (Figure 3D, D', black and yellow arrowheads). The proximal endoderm of the gill pouches began to express *Pax2/5/8a *while the distal portion of the gill pouches expressed *Pax2/5/8b *(Figure 3C, C', D', blue arrowheads). Presumptive dorsal precursor cells of the endostyle first began to express *Pax2/5/8a*, while presumptive ventral endostyle cells expressed *Pax2/5/8b *(Figure 4C, C', D, white arrowheads).

#### Late hatchling stages

By the late hatchling stage, organs are distinct and internal cavities have started to expand. At this stage, the gills and mouth have opened to the outside, and the heart and the ciliary rings of the gills have both begun to beat. The direct development of larvacean urochordates permits the tracing of gene expression domains in organ rudiments from hatchlings until fully mature adults. In contrast, in ascidian urochordates, many organs begin to develop only at metamorphosis, just as many chordate-specific characters disappear.

*Pax2/5/8a *expression in late hatchling stages overlaps *Pax2/5/8b *in some domains: in the distal part of the gills from the ciliary rings (cr) to the external gill opening (Figure 4, purple arrowheads), and, transiently, in the posterior trunk epidermis (Figure 4A, B, C, red arrowheads). Other domains are unique to each gene, and in several regions expression of the paralogs appears complementary. For example, while the lips of the mouth express *Pax2/5/8a *(Figure 4A, C, black arrowheads), the pharynx just internal to the lips strongly expresses *Pax2/5/8b *(Figure 4B, D, black arrowheads); while dorsal cells of the endostyle express *Pax2/5/8a *(Figure 4A, C, white arrowheads), ventral cells of the endostyle express *Pax2/5/8b *(Figure 4B, D, white arrowheads); and while the most posterior section of the rectum expresses *Pax2/5/8a *(Figure 4A, C, green arrowheads), the anus expresses *Pax2/5/8b *(Figure 4D, green arrowhead). Except for the distal part of the gills, the posterior pharynx exclusively expresses *Pax2/5/8b*, including the gill endoderm where the two gill pouches meet medially (Figure 4B, D, blue arrowheads). And, strikingly, only one layer of the two-ply heart, the pericardium, expresses *Pax2/5/8b *(Figure 4B', D', orange arrowheads); the muscular myocardium does not.

**Figure 5:**
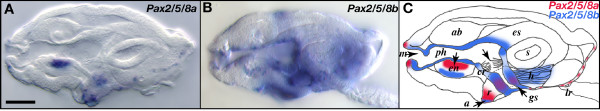
**Expression of larvacean *Pax2/5/8 *paralogs at pre-tailshift stage, when organogenesis nears completion**. (A) *Pax2/5/8a*. (B) *Pax2/5/8b*. (C) Schematic representation summarizing the non-overlapping *Pax2/5/8a *(red) and *Pax2/5/8b *(blue) expression domains. Arrows indicate perforations where epidermal fusions occur and *Pax2/5/8 *paralogs are expressed. Abbreviations: a, anus; ab, anterior brain; cr, ciliary ring; en, endostyle; es, esophagus; h, heart; lr, Langerhans receptor; m, mouth; ph, pharynx; r, rectum; s, stomach. Scale bar = 10 μm.

A subtle metamorphic event called 'tailshift', the rapid reorientation of the tail from a posterior to a ventral position with respect to the trunk, signals the beginning of the juvenile stage in *Oikopleura*. Just prior to tailshift, the two *Pax2/5/8 *paralogs continue to show mostly non-overlapping expression patterns, now in clearly differentiated organs. *Pax2/5/8a *(Figure 5A) is still expressed around the mouth (including ciliated sense organs in the upper lip), in the dorsal, iodine-binding corridor cells of the endostyle, in the gill endoderm, in the rectum, and in a row of cells at the posterior margin of the oikoplastic epithelium (a specialized secretory tissue that covers much of the trunk). Expression of *Pax2/5/8b *(Figure 5B) is also a continuation of domains identified earlier, including the pharynx and gills, the ventral endostyle, the anus, and the pericardium. New domains, however, now appear in dorsal, ventral and lateral fields of the oikoplastic epithelium, the tissue that will soon secrete the first filter-feeding 'house' the animal inhabits [61]. Figure 5C is a schematic diagram summarizing the expression domains of both *Oikopleura Pax2/5/8 *paralogs in late hatchlings. Table 1 summarizes by tissue and developmental stage all *Pax2/5/8 *expression domains.

**Table 1:** Summary of *Pax2/5/8 *expression domains during *Oikopleura *development

	**Tailbud**	**Early hatchling**	**Mid-hatchling**	**Late hatchling**
Stomodeum	a	a	a/b	a/b
Pharynx	a	a/b	a/b	b
Langerhans receptors	a	a+b	a	a
Endostyle			a/b	a/b
Gills			b	a/b
Anus				a/b
Posterior trunk epidermis				a+b
Heart				b

a, *Pax2/5/8a*; b, *Pax2/5/8b*; a/b, expression of paralogs does not spatially overlap or only partly overlaps; a+b, both paralogs spatially overlap.

## Discussion

These investigations of the structure and expression of *Pax2/5/8 *gene duplicates in *Oikopleura*, when analyzed in a comparative context with shared and independent *Pax2/5/8 *gene duplications in other chordate lineages, illuminate a variety of problems in the evolution of chordate developmental mechanisms and the principles that govern change in duplicated gene function over time. The work resolves alternative hypotheses for the homologies of the ascidian atrial siphon, illuminates evolution of the thyroid, identifies candidates for ancestral gene subfunctions, and defines the origin of functional innovations in this gene family.

### *Pax2/5/8 *and the evolution and development of the chordate heart

The shared expression of cardiac developmental genes (for example, *Nkx-2.5*, *dHand*, and *Mesp1*) supports the homology of bilaterian hearts from chordates to flies [62-69]. The evolutionary relationship of the single-chambered heart of non-vertebrate chordates to the individual chambers of the innovative multi-chamber vertebrate heart, however, is still unclear (reviewed in [70]). Our work reveals unprecedented *Pax2/5/8 *expression in the developing heart of a chordate, and may provide a late ontogenetic marker for assessing homology of tissue layers among simple, urochordate hearts.

In contrast to the single-layered heart of cephalochordates, urochordates have a two-layered heart. The simple heart of *Oikopleura dioica *consists of two epithelial layers lying just medial to the left stomach lobe. In *Oikopleura *species, the lateral wall of the heart contains the muscle fibers while the medial wall is a thin pericardial membrane (called 'procardium' in [71]). Peristaltic heart contractions, which periodically reverse, cause the hemolymph to course between the heart and the stomach wall [71,72]. In comparison, the ascidian heart rolls up to become tubular rather than planar, but also has a contractile layer and a non-muscular, pericardial layer (reviewed in [70]). The larvacean pericardial membrane, therefore, is the likely homolog of the ascidian pericardium.

In ascidians, heart development can be broadly divided in two phases (reviewed in [70]). In the first phase, heart cell precursors become fated during early cleavage and embryonic stages, and after a complex process of muscle cell migration from the tail to the trunk, heart development temporarily arrests. In the second phase, after metamorphosis, heart development re-initiates and after final differentiation, the heart starts to beat and becomes functional. In larvaceans, the transparency and absence of a drastic metamorphosis provides a complementary model system for the study of heart development. In *O. dioica*, the heart starts beating less than 24 hours post fertilization, before tailshift. In late larvacean hatchlings, when heart differentiation is at or nearing completion, the pericardium expresses *Pax2/5/8b*. In ascidians, however, neither *Pax2/5/8 *paralog has been shown to be expressed in the heart, perhaps because most published analyses of *Pax2/5/8 *expression in *Ciona*, *Halocynthia *and *Herdmania *[48,53,60,73,74] have not included late developmental stages after metamorphosis (3 to 4 days after fertilization). Therefore, whether *Pax2/5/8 *expression in the heart – and more specifically in the pericardium – is a shared feature among urochordates remains to be investigated.

Cephalochordates have a single-layered, muscularized blood vessel ventral to the gut that propels the hemolymph by peristalsis [75,76]. During amphioxus development, the primordium of this muscularized vessel expresses a homolog of vertebrate *NK2 *class genes – a group that includes several genes necessary for vertebrate cardiac development – suggesting that the amphioxus vessel is homologous to the vertebrate heart [69]. *Pax2/5/8 *is not reported to be expressed in the circulatory vessels of amphioxus [52], and likewise, amphioxus has no structure that appears to be morphologically comparable to the *Pax2/5/8*-expressing larvacean pericardium or to the pericardia of ascidians or vertebrates.

In vertebrates, mouse *Pax2 *and *Pax5 *are not expressed in the heart [77,78], and although *Pax8 *expression has been detected in the heart of newborn mice, its level was equal to or less than all other tissues tested except kidney [79], suggesting that it may represent background levels. No role for *Pax2, Pax5*, or *Pax8 *has been demonstrated in the heart in knockout mutations in mouse [80-82]. Consistent with these results, our survey of EST databases in NCBI  reveals the absence of *Pax2/5/8 *genes in the heart transcriptomes of human, mouse, frogs and zebrafish. Thus, we can infer that *Pax2/5/8 *expression in the heart was either already present in the last common ancestor of olfactores (that is, urochordates + vertebrates) and lost in the vertebrate lineage, or was a neofunctionalization event within the urochordates, and perhaps within larvaceans.

### Evidence of *Pax2/5/8 *subfunction partitioning in thyroid homologs

**Figure 6:**
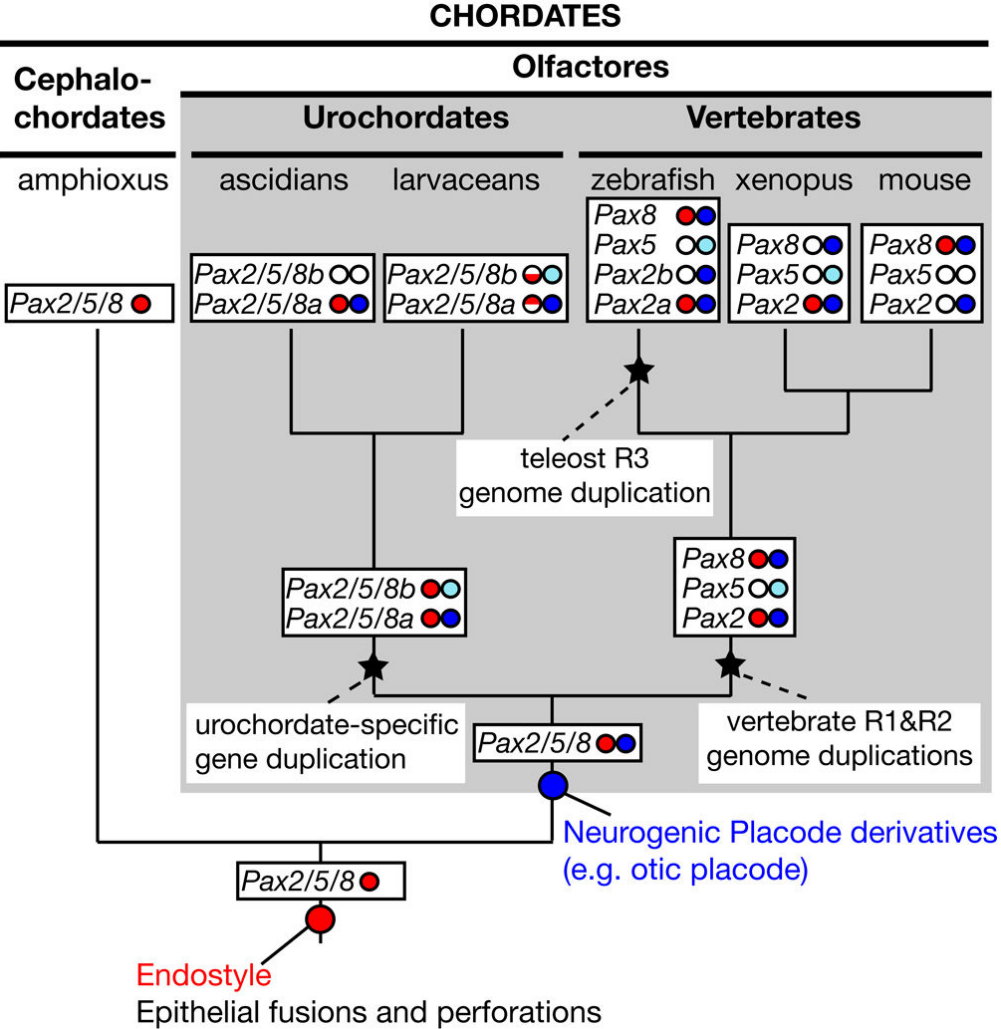
**Hypothesis for the evolution of chordate Pax2/5/8 subfunctions**. In stem chordates, Pax2/5/8 already played a role during the development of the endostyle, the homolog of the vertebrate thyroid, as well as in controlling genes for making epithelial fusions and perforations (for example, gill openings). The Pax2/5/8 subfunction related to the development of neurogenic placodes (for example, otic placode) appears to be restricted to the olfactores; like vertebrate orthologs, urochordate *Pax2/5/8a *genes are expressed from just after neurula stage in paired thickenings overlapping the early expression of other placode-marking genes [39]. Inferred origins for some of the functions (circles in stems) performed by the pleiotropic Pax2/5/8 gene family and inferred subfunction partitioning are schematized in the context of the chordate phylogeny. Gene duplication events (stars) in different chordate lineages permitted independent partitioning of endostyle/thyroid (red circles) and otic placode (blue circles) subfunctions among gene paralogs. Half semicircles denote dorsal and ventral expression domains. Pale blue circles denote paralogs whose expression is inferred to have become delayed, transient or spatially narrowed in development of the otic system. White circles indicate inferred subfunction losses. [43,45,48,52,56,73,84,85,118,119].

The thyroid is an endocrine gland located in the neck ventral to the pharynx in humans and other vertebrates, and it regulates energy production and growth by synthesizing tyrosine-based, iodine-containing hormones (T3 and T4). The presumed homolog of the thyroid in filter-feeding, non-vertebrate chordates is the endostyle, an organ located ventral to the pharyngeal floor that functions in iodine binding and secretion of food-trapping mucus [83]. The homology between the thyroid and the endostyle is further supported by the expression of similar molecular markers, including members of the Pax2/5/8 family ([84] and references therein). Our results show that the larvacean endostyle expresses *Pax2/5/8 *genes in a pattern similar to that described for amphioxus [52], ascidians [84], and vertebrates [56,85]. The last common ancestor of all three chordate subphyla, therefore, probably employed a *Pax2/5/8 *gene in the development of an endostyle-like organ that evolved into the endostyle of cephalochordates and urochordates and the thyroid of vertebrates (Figure 6).

*Pax2/5/8 *subfunctions related to vertebrate thyroid development have likely suffered independent processes of subfunction partitioning among paralogs (Figure 6). For instance, in mouse, *Pax8 *is expressed during thyroid development but *Pax2 *is not [56,85]; in frogs, however, subfunctions of these genes are reversed, as *Pax2 *but not *Pax8 *is involved in the development of the thyroid [43]. These results suggest that ancestral vertebrate thyroid regulatory subfunctions of *Pax2/5/8 *were preserved by both *Pax2 *and *Pax8 *in stem amniotes, and resolved independently in the amphibian and mammalian lineages. In zebrafish, both *pax8 *and *pax2a *(previously called *pax2.1*) retain thyroid expression, although they are activated at different times in development [56].

Although the duplication of *Pax2/5/8 *paralogs in urochordates occurred before the divergence of the larvacean and ascidian lineages [38] (Additional File 1), the endostyle of *Oikopleura *expresses both *Pax2/5/8 *paralogs (*Pax2/5/8a *dorsally, and *Pax2/5/8b *ventrally), while the endostyle of ascidians does not express *Pax2/5/8b*, but expresses *Pax2/5/8a *in both the dorsal and ventral domains, the sum of the pattern for the two *Oikopleura *duplicates [84]. These lineage-specific differences in expression patterns of orthologous *Pax2/5/8 *duplicates suggest that in the last common ancestor of larvaceans and ascidians, both *Pax2/5/8a *and *Pax2/5/8b *were expressed both dorsally and ventrally, and that after lineages diverged, in larvaceans the dorsal and ventral endostyle subfunctions partitioned to different *Pax2/5/8 *genes, but in ascidians both subfunctions remained associated with the *Pax2/5/8a *paralog and both were lost by the *Pax2/5/8b *paralog. As vertebrate Pax8 can directly bind the promoters of thyroid follicle cell-specific genes such as thyroperoxidase [86], we raise the hypothesis that urochordate *Pax2/5/8a *genes, which are expressed in peroxidase-producing, dorsal cells of the endostyle [87,88], play a homologous role while urochordate *Pax2/5/8b *genes have lost this function.

### Evidence of *Pax2/5/8 *subfunction partitioning in the anterior pharynx and stomodeum

Comparison between ascidians, larvaceans and cephalochordates suggests that stem chordates expressed *Pax2/5/8 *in the stomodeum and anterior pharynx, but that this expression domain was lost in the vertebrate lineage. Larvaceans and ascidians both have ciliated mechanosensory organs that ring the mouth and are involved in a filter-feeding, particle-rejection response [39,89,90]. Although the oral sensory cell types themselves are morphologically different in the two urochordate classes, these organs may share a common origin in a stomodeal sensory placode that has been lost in the vertebrate lineage [39,73,90-94]. Although both larvacean *Pax2/5/8 *paralogs are expressed in this stomodeal domain, their patterns are distinct: *Pax2/5/8a *expression in the rostral pharynx of early hatchlings is replaced with *Pax2/5/8b *by mid-hatchling stages. In mid-hatchlings, *Pax2/5/8a *expression is in the external ectoderm surrounding the mouth opening, including the two bristle-bearing cells of the upper lip, while *Pax2/5/8b *is expressed just inside the mouth, including the ciliated sensory cells of the circumoral organ. Although the two mechanosensory cell types fall into expression domains of different *Pax2/5/8 *paralogs, they are innervated by the same branched sensory axons emanating from a pair of rostral brain cells [39,89].

The single amphioxus *Pax2/5/8 *gene is expressed both externally around the developing mouth and internally in the pharynx [52]; this expression domain corresponds to the sum of expression domains occupied by different larvacean paralogs, as expected by subfunction partitioning [2] if the last stem chordate had separate regulatory elements governing the contiguous external and internal pharyngeal domains that partitioned to different larvacean paralogs after the *Pax2/5/8 *duplication event. Ascidian *Pax2/5/8a *is expressed in the invaginating stomodeum but *Pax2/5/8b *is expressed in the buccal cavity, a pattern comparable with the late hatchling expression domains of the *Oikopleura *orthologs. At least some subfunctions, therefore, appear to have partitioned between *Pax2/5/8 *paralogs before the larvacean and ascidian lineages diverged. The ascidian *Pax2/5/8a *gene, however, appears to lack expression comparable with the early, internal expression of *Oikopleura Pax2/5/8a *in the rostral pharynx, a difference that may derive from the developmental delay and incomplete development of endodermal organs experienced by ascidian larvae compared with the uninterrupted and complete endodermal ontogeny in larvaceans.

### *Pax2/5/8 *expression and the Langerhans receptor, ascidian atrial siphon, and vertebrate otic placode

The expression of *Pax2/5/8 *genes in the Langerhans receptors of *Oikopleura *helps us understand apparently conflicting interpretations of the ancestral role of *Pax2/5/8 *in the origin of the vertebrate otic placode. Previous evidence supports the notion that larvacean Langerhans receptors are homologous to hair cell-like sensory organs in the ascidian atrium, the chamber surrounding the branchial basket [39,73,95]. Expression of *Pax2/5/8a *in the ascidian atrium and expression of *Pax2 *and *Pax8 *in the vertebrate hair cell-producing otic placode suggested the hypothesis that the atrium of ascidians is homologous to the otic vesicles of vertebrates [48].

An alternative to the 'placode hypothesis', however, arose from the finding that in amphioxus, the developing gill slits and mouth express *Pax2/5/8*. This alternative suggests that *Pax2/5/8 *expression in the atrial primordium of ascidians reflects an ancient gene function in the perforation, adhesion and fusion of epithelial layers, such as in the epithelia of gill openings, rather than the homology of atrial primordia and vertebrate placodes [52]. The 'epithelial fusion hypothesis' is consistent with the finding that among vertebrates, *Pax2 *apparently plays a role in gill slit perforation in *Xenopus *[96] and *Pax8 *is necessary for vaginal opening in mouse [97]. Furthermore, *Pax2 *and *Pax8 *regulate genes that control the composition of the extracellular matrix in epithelial fusions [42,98].

Evidence to resolve this dilemma comes from examination of gene expression and organ structure in a phylogenetic context. Our analysis of larvacean *Pax2/5/8 *expression teases apart epithelial fusion from placode formation and suggests that *Pax2/5/8 *likely functioned in both processes in ancient chordates. In *Oikopleura*, several *Pax2/5/8 *expression domains can be grouped into two overlapping categories: sites of epithelial fusion and sites of sensory cell development. At the sites of epithelial fusion, larvacean *Pax2/5/8 *paralogs are expressed at the junction of the gill pouch with the epidermis, the joining of the rectum to the epidermis at the anus, and the fusion of the stomodeum and pharynx at the mouth. At sites of sensory cell development, *Oikopleura's Pax2/5/8 *paralogs are expressed at the stomodeal placode and the putative acousticolateralis placode homolog (the Langerhans organ primordia). Therefore, in contrast to ascidians, in which hair cell-like organs and sites of perforation are conflated in the atrium [95], *Oikopleura*'s paired mechanosensory organs are topographically separate from the gill openings.

In agreement with the perforation argument [52,99], expression of *Oikopleura Pax2/5/8 *as in amphioxus, in the gill openings and anus, which lack sensory cell types, supports the hypothesis of an ancient function for *Pax2/5/8 *in the adhesion and fusion of epithelial layers and in the promotion of epithelial perforations. On the other hand, expression of *Oikopleura Pax2/5/8a *at early tailbud stage in the primordia of the Langerhans organs occurs long before the fusion of the gill endoderm with the ectoderm, which happens at the late hatchling stage. This timing gap argues against the interpretation that early expression of *Pax2/5/8 *in the Langerhans domain is associated only with remodeling the extracellular matrix for gill perforation. Instead, early *Pax2/5/8 *expression defines the location of paired, thickened sensory organ primordia, in agreement with the hypothesis of Wada et al. [48] that *Pax2/5/8 *marks a urochordate placode. Larvacean *Pax2/5/8 *expression adds to a growing body of morphological and gene expression data that supports the origin of cranial placodes in early chordates, rather than in vertebrates, as had been long assumed [39,48,73,93-95], [100-105], and strengthens the case for an early origin specifically of the otic or acousticolateralis placode.

Therefore, analysis of *Pax2/5/8 *paralogs in *Oikopleura *has allowed us to reconcile the 'placode hypothesis' and 'epithelial fusion hypothesis', supporting an evolutionary scenario in which the role of *Pax2/5/8 *in epithelial fusions and perforations was already present in stem chordates, and the role of *Pax2/5/8 *related to placode development is likely also ancient, though perhaps restricted to Olfactores (Figure 6).

### Evolution of *Pax2/5/8 *genetic pathways

*Pax, Eya, Six *and *Dach *genes form a genetic network in several biological processes, including in sensory placode development (reviewed in [81,106]). *Pax2*, *Pax5*, *Pax8*, *Eya1*, *Six1*, and *Dach1 *are co-expressed during fish otic vesicle development (see for example [107,108]), where they interact to specify otic tissue and maintain its continued ontogenesis. Larvacean *Pax2/5/8 *genes are expressed in presumptive sensory tissues previously reported also to express *Eya *and *Six *orthologs [39], namely around the mouth and in the mechanosensory Langerhans receptors. The two *Pax2/5/8 *paralogs may interact with different subsets of *Eya-Six-Dach *genes in different tissues. For instance, in developing sensory tissues, *Pax2/5/8a *expression in tailbud and early hatchling stages is similar to that of *Eya *and *Six3/6a *in the developing Langerhans receptor primordia, and to *Six1/2 *and *Six3/6a *expression around the mouth, including sensory cilia-bearing cells of the upper lip. Endodermal *Pax2/5/8b *expression, on the other hand, most strongly overlaps that of *Six3/6a *in the rostral pharynx and *Eya *in the gill endoderm in mid-hatchling stages. Similarly, ascidian *Pax2/5/8a *expression overlaps *Eya *and *Six1/2 *in the atrial primordia and *Eya*, *Six1/2*, and *Six3/6 *in the stomodeal domain, but *Pax2/5/8b *exhibits broad expression in the ectoderm and may also overlap *Eya*, *Six1/2*, and *Six3/6 *in a way that larvacean *Pax2/5/8b *does not [73]. Such differences between presumed *Pax2/5/8 *paralogs and orthologs is further evidence that, though the *Pax-Six-Eya-Dach *'module' is conserved at the level of gene families, paralogs within each module may differ both within a developmental program and between lineages [39,99]. Not surprisingly, then, sequence analysis shows that several known protein interaction domains differ between the larvacean *Pax2/5/8 *paralogs and between the larvacean and ascidian orthologs (Figure 1), consistent with divergence of the molecular pathways in which these Pax proteins participate.

### *Pax2/5/8 *gene duplications and the evolution of gene functions

A requirement for partitioning of ancestral gene functions is that the ancestral gene must have multiple *independently mutable *functions either in protein-coding domains or in regulatory elements that drive restricted expression: in other words, units that are by definition 'subfunctions'. Direct evidence for independently mutable *Pax2/5/8 *subfunctions in an extant gene comes from the *Drosophila *ortholog, called *D-Pax2*, in which mutations in separate regulatory elements affect development of either ommatidia or sensory bristles [109]. The known functions of vertebrate *Pax2/5/8 *genes are diverse and include the establishment of the midbrain-hindbrain border [110-113], specification and mature function of thyroid follicular cells [85], specification and morphogenesis in the pronephros [80], differentiation of interneuron subtypes in the central nervous system (see for example [114]), promotion of correct axon guidance in the optic nerves [115], and morphogenesis and sensory cell specification in the epibranchial, otic and lateral line sensory placodes (see for example [96]). It is not known, however, how many of these functions, segregated among extant genes, resulted from independently mutable ancestral subfunctions.

From the partially overlapping expression patterns of vertebrate *Pax2/5/8 *genes, we can infer that subfunction partitioning has occurred in this gene family. Next, comparative analysis of expression domains in the chordate phylogenetic context helps us infer when subfunctions arose. Parallel, independent partitioning of *Pax2/5/8 *endostyle/thyroid functions in both vertebrate and urochordate lineages suggests that the single *Pax2/5/8 *gene of stem olfactores had already acquired independently mutable regulation for endostyle/thyroid expression. Likewise, paralleling segregation of mammalian otic placode subfunctions to *Pax2 *and *Pax8 *[42,45], larvacean otic placode-like tissues express only one *Pax2/5/8 *paralog early and in a pattern overlapping orthologs of other vertebrate placode markers of the *Six *and *Eya *gene families [39] (Figure 6). It remains possible, though, that despite the similarity between vertebrate and urochordate *Pax2/5/8 *gene expression patterns, each lineage separately evolved independently mutable functions in the endostyle/thyroid and otic placode development and that the ancestral gene did not already bear these subfunctions.

Within the urochordate lineage, further parsing of gene functions may represent subfunctions that were present in the ancestral urochordate *Pax2/5/8 *gene. For example, at some sites of epithelial fusion, larvacean *Pax2/5/8 *paralogs exhibit complementary expression, for example just outside (*Pax2/5/8a*) or just inside (*Pax2/5/8b*) the mouth and just outside (*Pax2/5/8b*) or just inside (*Pax2/5/8a*) the anus. Therefore, though a role in epithelial fusions is probably ancient in Chordata, urochordate paralogs may have taken on separate refinements of the same function.

In addition to the partitioning of ancestral functions, we might also expect to detect apparent neofunctionalizations of gene duplicates, particularly when we compare deeply diverging lineages such as vertebrates and urochordates. The role of vertebrate *Pax5 *in the B-lymphoid lineage of the immune system might be one example of a vertebrate neofunctionalization [55]. Similarly, because no comparable expression can be found outside of Urochordata, a role for *Pax2/5/8b *in the pericardium of the heart could be a novel deployment of *Pax2/5/8 *exclusive to the urochordates or even to the larvacean lineage.

Though observed *Pax2/5/8 *expression patterns are consistent with a hypothesis of ancestral subfunction partitioning, this analysis detects only transcriptional regulatory differences and might underestimate the extent of actual subfunction partitioning. For example, mRNA expression of the paralogs may overlap in a given tissue, but the structurally different proteins the genes encode may not have redundant functions in those tissues. In addition, post-transcriptional regulation would also be missed in our analysis. Partitioned genes likely continue to evolve after their initial partitioning, and such divergence could complicate distinguishing subfunctionalization from neofunctionalization when comparing gene functions in deeply diverging lineages. Nonetheless, functional analysis of urochordate *Pax2/5/8 *genes could help distinguish between ancestral chordate subfunction partitioning and convergent patterns of neofunctionalization. For example, if Pax2/5/8 proteins carry out a similar function in the vertebrate thyroid as in the urochordate endostyle, this would be more concrete evidence that an ancient function was partitioned to paralogs in both chordate subphyla.

## Conclusion

The present work shows how analyzing the evolution of gene families that have experienced multiple independent gene duplication events in related lineages can improve understanding of the evolution of the genetic mechanisms underlying the development of homologous structures of anatomically divergent organisms (for example the endostyle of non-vertebrate chordates and the thyroid of vertebrates), and can help to identify gene subfunctions that otherwise may be difficult to recognize because of extensive pleiotropy (for example, Pax2/5/8 subfunctions in epithelial fusions around perforations and development of placode derivatives). Analysis of the evolution of subfunctions in a phylogenetic context identifies lineage specific innovations (for example, placode derivative homologs in olfactores). The modular fashion in which gene subfunctions partition after independent gene duplication events in various lineages implies the existence of independent regulatory elements that control temporal and spatial expression for each subfunction. For instance, comparison of *Pax2/5/8 *expression patterns in the endostyles of amphioxus, *Oikopleura*, and ascidians predicts the presence of separable regulation for expression in the iodine-binding dorsal component and the supporting ventral component of the endostyle/thyroid, a hypothesis that remains to be tested. The identification of the complete set of gene orthologs and paralogs in gene families in an increasing number of completely sequenced genomes will likely reveal additional cases of independent gene duplications and subfunctionalization events, the analysis of which will improve our understanding of the evolution of gene functions and the implications in the evolution and diversity of living forms.

## Methods

### Biological materials

*Oikopleura dioica *animals were collected in the Pacific Ocean near the Oregon Institute of Marine Biology (Charleston, OR), and were cultured in the laboratory at the University of Oregon (Eugene, OR, USA) at 13°C in 10 μm-filtered seawater for several generations. The transparency of *Oikopleura *embryos and adults allows non-invasive study of internal anatomy at the level of individual cells and the tracing of organs from embryo to adult. For some images, we merged DIC optical sections using Adobe Photoshop software to integrate images of structures that spanned multiple focal planes.

### Whole-mount in situ hybridization

Whole-mount in situ hybridization was performed as described [116,117] with minor modifications. Fixed, dehydrated embryos were dechorionated manually with glass needles before re-hydration; to reduce background, Tween-20 concentration was increased from 0.1% to 0.15% in the hybridization buffer, PBT solution and post-hybridization washing buffers. Embryos were mounted in 80% glycerol for microscopy. Riboprobes for detecting the expression of *Pax2/5/8a *and *Pax2/5/8b *(Genbank accession numbers, respectively: AY870648, AY870649) genes are described in [38].

## List of abbreviations

DDC: duplication-degeneration-complementation.

## Authors' contributions

SB identified, cloned and characterized the expression pattern of *Oikopleura Pax2/5/8b*, performed part of the comparative analysis and wrote part of the manuscript. CC identified, cloned and characterized the expression pattern of *Oikopleura Pax2/5/8a*, performed part of the comparative analysis and wrote part of the manuscript. JHP supervised the project, performed part of the comparative analysis and wrote part of the manuscript. All authors read and approved the final manuscript.

## Supplementary Material

Additional file 1 Phylogenetic relationships among chordate Pax2/5/8 proteins. The data provided illustrates the independent gene family expansions that permitted parallel histories of subfunction partitioning among vertebrate paralogs (Pax2, Pax5 and Pax8) and among urochordate paralogs (Pax2/5/8a and Pax2/5/8b). Click here for file
