# Supplementary material for: Evolution of developmental roles of Pax2/5/8 paralogs after independent duplication in urochordate and vertebrate lineages
Source: BMC Biol. 2008 Aug 22;6:35. doi: 10.1186/1741-7007-6-35 (PMC2532684; doi:10.1186/1741-7007-6-35)
Supplement: Additional file 1 — Phylogenetic relationships among chordate Pax2/5/8 proteins. The data provided illustrates the independent gene family expansions that permitted parallel histories of subfunction partitioning among vertebrate paralogs (Pax2, Pax5 and Pax8) and among urochordate paralogs (Pax2/5/8a and Pax2/5/8b). [file 1741-7007-6-35-S1.pdf]

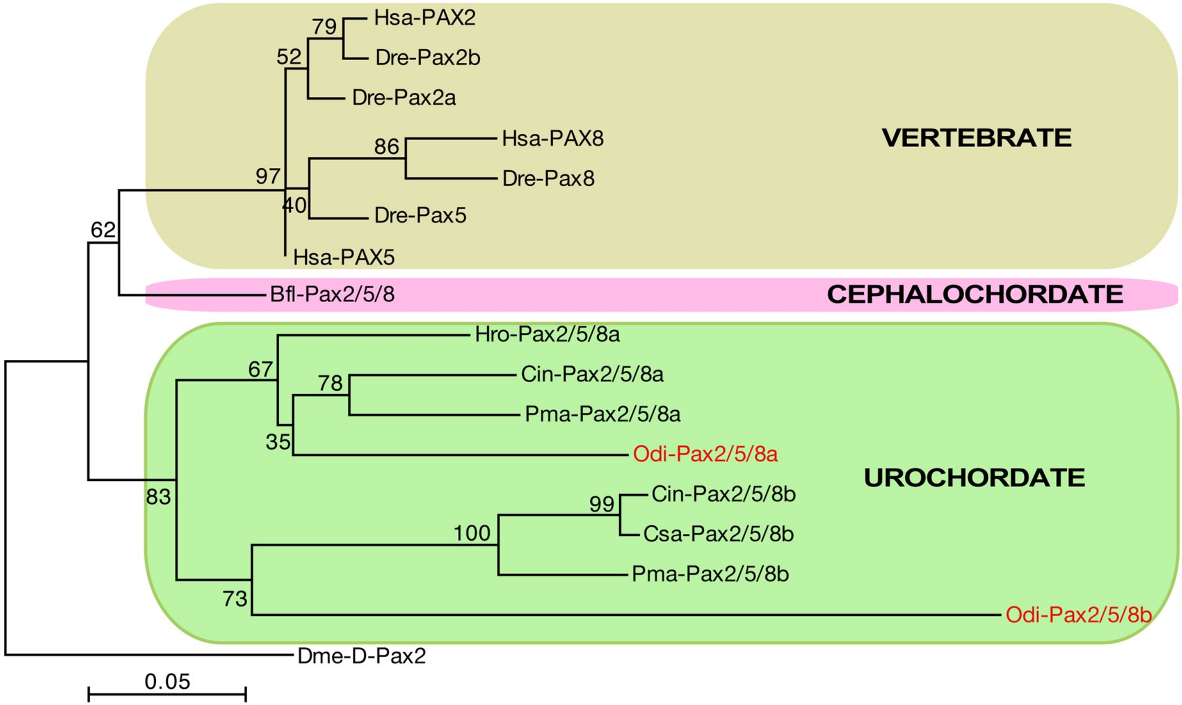

Supplementary figure. Phylogenetic relationships among chordate Pax2/5/8 proteins, illustrating the independent gene family expansions that permited parallel histories of subfunction partitioning among vertebrate paralogs (Pax2, Pax5 and Pax8) and among urochordate paralogs (Pax2/5/8a and Pax2/5/8b). Protein alignment and phylogenetic analysis was performed as described in reference [38]. Vertebrates: Dre, *Danio rerio;* Hsa, *Homo sapiens*; Cephalochordates: Bfl, *Branchiostoma floridae*; Urochordates: Ascidians: Cin, *Ciona intestinalis*; Csa, *Ciona savignyi*; Hcu, *Herdmania curvata*; Hro, *Halocynthia roretzi*; Pma, *Phallusia mammillata*; and Larvaceans: Odi, *Oikopleura dioica (red)*; Protostomes: Dme, *Drosophila melanogaster*.
